# Comparative Analysis of Regional Blood-Brain Barrier Permeability in Humans and Rodents: An Evolutionary Perspective

**DOI:** 10.21203/rs.3.rs-9869051/v1

**Published:** 2026-06-15

**Authors:** Sheida Mirloo, Lyna Kamintsky, Shayna Cort, Jamil Muradov, Saara Mansoor, Laith Alhadeed, Alaa Abu Ahmad, Basim Shafiq, Amirhossein Talebanpour, Rotem Saar -Ashkenazy, Yonatan Serlin, Alon Friedman

**Affiliations:** Dalhousie University; Dalhousie University; Dalhousie University; Dalhousie University; Dalhousie University; Dalhousie University; Dalhousie University; Dalhousie University; Dalhousie University; Ashkelon Academic College; Dalhousie University; Dalhousie University

**Keywords:** Blood-brain barrier, DCE-MRI, BBB permeability, Regional heterogeneity, Aging, Sex differences, Plasma volume fraction

## Abstract

**Background:**

The blood-brain barrier (BBB) serves a critical function in regulating transport between the blood and the brain, with its dysfunction being implicated in numerous neurological diseases such as neurodegenerative disorders, epilepsy, stroke, traumatic brain injury, and brain tumors. Understanding the regional heterogeneity in BBB properties in healthy individuals remains limited. This study aims to characterize regional BBB permeability variations in the healthy brain using dynamic contrast enhanced magnetic resonance imaging (DCE-MRI) and explore differences related to sex and age.

**Methods:**

We retrospectively analyzed DCE-MRI scans from 102 healthy participants aged 19–85 (55.8% female), assessing BBB permeability to a Gadolinium-based contrast agent and plasma volume fraction across 124 brain regions. We examined the effects of sex and age on BBB permeability and plasma volume fraction. Additional studies were conducted on 11-week-old Sprague Dawley rats using DCE-MRI and fluorescent microscopy following peripheral injection of Evans blue dye.

**Results:**

We show that BBB permeability varies across brain regions, being lower in more recently evolved cortical regions, such as the frontal cortex, compared to the phylogenetically older brainstem and subcortical areas. Plasma volume fraction showed the opposite trend with higher vascularization in cortex compared to brainstem. Age-related analysis revealed an increase in BBB permeability (normalized to plasma volume fraction) that was more pronounced in males than females.

**Conclusions:**

This study underscores an evolutionary perspective in BBB properties, highlighting a gradient from higher permeability in evolutionarily older brain regions (e.g. brainstem) to lower permeability in evolutionarily younger regions (e.g. frontal cortex). These permeability differences accentuate with age, particularly in males.

## Background

The blood-brain barrier (BBB) regulates the transport of cells, molecules, and ions between the blood and the brain (1, 2). Proper function of the BBB is critical for normal brain function (3). The growing recognition of BBB dysfunction in the pathogenesis and progression of neurological disorders has highlighted the importance of developing means to measure BBB integrity in living humans. To this day, dynamic contrast-enhanced magnetic resonance imaging (DCE-MRI) remains the most common approach to quantify BBB integrity and other vascular features, including plasma volume (v ) fraction (4–7). While most studies focused on exploring vascular properties under pathological conditions (5, 7–11) as well as normal aging (12, 13) data on regional variability and the effect of physiological factors including age and sex remain limited. The aim of the current study is to characterize regional differences in BBB permeability and vascularization in the healthy human brain and explore differences associated with sex and age.

## Methods

### Human data

This study was approved by the Ethics Committee of Soroka Medical Center, Beer Sheva, Israel. For the present study we analyzed 102 DCE-MRI scans from healthy volunteers, recruited for previous studies (10, 11, 14). All participants signed informed consent forms. The inclusion criteria were ages between 18 and 85 years and normal kidney function. Exclusion criteria included a history of significant brain trauma, neurological or psychiatric disorders, or contraindications for MRI. Participants were divided into two age-based groups (Table 1): 35 participants aged 19–30 were considered as the “young” group. The effect of age and sex was analyzed across all participants.

### MRI acquisition in human subjects

MRI scans were acquired using a 3T Philips Ingenia scanner. The sequences acquired for BBB measurement were: (i) a T1-weighted anatomical scan (3D gradient echo, TE/TR = 3.7/8.2 ms, acquisition matrix 432 × 432, voxel size: 0.5 × 0.5 × 1 mm); (ii) a 3D T1-weighted-FFE sequence (TE/TR = 2/10 ms, acquisition matrix: 256 × 256, voxel size: 0.89 × 0.89 × 6 mm, flip angles: 10, 15, 20, 25 and 30°) for the calculation of pre-contrast longitudinal relation time *T*_1−0_; and (iii) a T1 weighted-FFE 3D axial DCE-MRI scan (TE/TR = 2/4 ms, acquisition matrix: 192 × 187 (reconstructed to 256 × 256), voxel size: 0.9 × 0.9 × 6 mm, flip angle: 20°, Δt = 10 sec, temporal repetitions: 100) acquired between minutes 6 to 16 after an intravenous bolus injection of 0.1 mmol/kg of gadoterate meglumine (Gd-DOTA, Dotarem, Guerbet, France) using an automatic injector at a rate of 1.5 ml/sec.

### DCE-MRI analysis of human data

BBB permeability analysis was performed using the BBBdetect software (7, 11, 15, 16). In brief, images undergo registration and normalization to MNI coordinates using SPM12 (www.fil.ion.ucl.ac.uk/spm). Next, the MR signal intensities are converted to values of contrast concentration (17) and the concentration of each voxel over time is represented as concentration-time curves. Analysis of each pixel’s time curve is used to determine whether contrast washes out from the tissue over time or accumulates in the tissue due to BBB leakage. Specifically, the slope of a linear regression analysis is used to measure the rate of contrast washout/accumulation (i.e., BBB permeability, mMol/min) (11, 18). To mitigate inter-individual variabilities such as heart rate, blood flow, or variations in contrast flow rates, each voxel’s slope is normalized to the slope of the superior sagittal sinus (10, 11). For regional BBB permeability analysis, each scan is segmented to 124 brain regions based on the MNI brain atlas (https://guthub.com/neurodebian/smp12/tree.master/tpm) (Additional Fig. 1).

BBB permeability is quantified using the Veksler Model (11). For regional analysis, ten anatomically defined brain regions, referred to as “lobes,” were examined (Additional Fig. 1). Normalized BBB permeability was computed for each individual by dividing each lobe’s permeability value by the corresponding brainstem value.

Since BBB permeability, measured as the accumulation of a Gadolinium-based (Gd-based) contrast agent, may be influenced by vascular density, we also calculated fractional plasma volume (v ) using the Extended Tofts Model. v values were constrained to the physiologically plausible range (0–1). Voxels yielding estimates outside this range were excluded, as such values are non-physical and typically arise from model instability, noise, or motion artifacts (19). Similar to BBB permeability, normalized v is obtained by dividing each lobe’s v value by the v measured in the brainstem.

Lobe-specific thresholds for abnormal BBB permeability are determined based on the sum of the median and two median absolute deviations (MAD) of lobe-specific values in healthy young individuals (Group A). Whole-brain BBB permeability is measured as the percent of brain voxels with suprathreshold permeability values.

3D human brain images are generated using BrainNetViewer GUI (20).

### Brain volume analysis of human data

Volumetric analysis was performed on T1-weighted anatomical scans using the Volbrain pipeline (21).

### MRI acquisition and analysis in rats

All procedures were conducted in accordance with the guidelines of the Canadian Council on Animal Care and approved by the Dalhousie University Committee on Laboratory Animals. Eleven-week-old male Sprague Dawley rats (Charles River, Montréal, QC, Canada) were anesthetized with 2% isoflurane and scanned using a 3T Philips Ingenia scanner. The acquisition protocol included: (i) a FLASH T1-weighted sequence, acquisition matrix 108 × 108 (38 mm × 38 mm), 16 slices (1.2 mm), TR = 6.03 ms, TE = 2.98 ms, flip angle: 20°, 24 averages, 2 volumes; (ii) injection of 0.45 ml Multihance (as the CA) via a tail vein cannulation; and (iii) acquisition of a FLASH T1-weighted sequence, acquisition matrix 108 × 108 (38 mm × 38 mm), 16 slices (1.2 mm), TR = 6.03 ms, TE = 2.98 ms, flip angle: 20°, 24 averages, 8 volumes, ΔT = 3ms.

**Figure 1:**
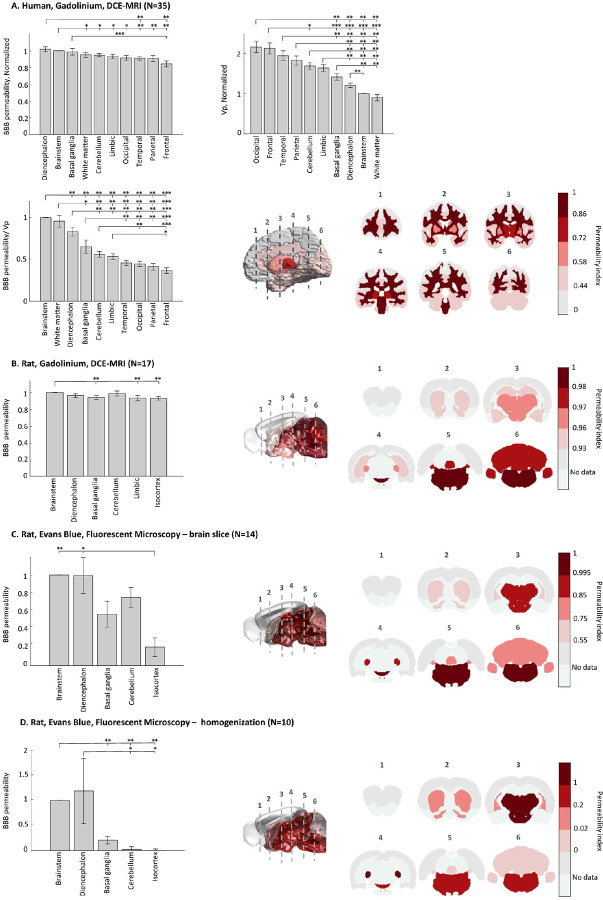
Regional BBB Differences in Healthy Human and Rodent Brain. Differences in regional BBB permeability were assessed in three groups: A. healthy individuals who underwent DCE-MRI scanning (n = 35, 26.22 ± 3.1 years, 48.6% female); Upper Left: median normalized BBB permeability values in the 10 clusters of regions (“lobes”). Upper Right: median normalized fraction plasma volume (v) differences between the lobes. Lower left: median normalized BBB permeability measurements/ normalized v. Lower middle: the sagittal brain slice represents the normalized BBB permeability/ normalized v. Darker red represents higher BBB permeability/v. Lower right: the color bar represents permeability values, ranging from gray (low permeability) to dark red (high permeability), corresponding to the permeability index of each region on six coronal brain slices moving from anterior to posterior regions of the brain. B. naïve rats that underwent DCE-MRI scanning (n = 17, 9–10-month-old males). C. brain slices from naïve rats that underwent histopathological analysis of Evans blue staining (n = 14, 9–10-month-old males) and D. homogenized brain tissue from naïve rats that underwent histopathological analysis of Evans blue staining (n = 10, 9–10-month-old males). Figures in the middle and right sections show the normalized BBB permeability values on rat brain slices. Darker red represents higher values. Mann-Whitney U test was used for statistical analysis. * p < 0.05, ** p < 0.001 and *** p < 0.0001 after adjustment for multiple comparisons. FDR correction was applied using q = 0.005. BBB: blood-brain barrier; DCE-MRI: dynamic-contrast-enhanced magnetic resonance imaging; FDR: False discovery rate.

Images were analyzed with an in-house MATLAB version R2023b script, as previously described (11). In brief, images were registered, and each voxel was represented by a curve of signal intensity over time. The slope of each voxel’s curve was calculated using a linear regression model to measure BBB permeability. Images were segmented into 6 lobes based on a rat brain atlas (22) (Fig. 1B and Additional Fig. 2). 3D mouse brain images were produced using BrainMesh GUI (https://github.com/BrainMesh/blob/master).

### Rat brain slicing and fluorescent microscopy analysis

All procedures were conducted in accordance with the guidelines of the Canadian Council on Animal Care and approved by the Dalhousie University Committee on Laboratory Animals. Eleven-week-old male Sprague Dawley rats (Charles River, Montréal, QC, Canada) were anesthetized with 3.5% isoflurane mixed with oxygen at a flow rate of 2L/min for a duration of 2 minutes. A 2% Evans Blue solution was injected into the tail vein at a dose of 2 mL/kg. Thirty minutes after the injection, the rats were anesthetized with 100 mg/kg sodium pentobarbital and perfused with buffered 0.9% NaCl pH 7.4. The brains were removed and submerged in 4% paraformaldehyde (PFA) for fixation. Each brain was cut into slices of 2mm thickness using the Alto Acrylic 1mm Rat Brain Coronal brain blocker. Brain images were acquired using Zeiss Axiocam 503 monofluorescent microscope with Zen 2.3 Lite software with two channels set at 540 nm and 680 nm wavelengths.

### Rat brain homogenization and fluorescent microscopy analysis

All procedures were conducted in accordance with the guidelines of the Canadian Council on Animal Care and approved by the Dalhousie University Committee on Laboratory Animals. Evans Blue dye (EBD) solution (2% in 0.9% saline) was prepared and administered to anesthetized rats (isoflurane: 1–2%, 99%, 1 L/min O_2_) via lateral tail vein injection (2 mL/kg body weight). Successful administration was confirmed by visible color change in the eyes and peripheral tissues. After 45–60 minutes of circulation, animals were euthanized with sodium pentobarbital (150 mg/kg) and transcardially perfused with 0.9% saline.

Brains were collected and dissected into regions of interest. Tissue samples were homogenized following the protocol described by Wang et al. (2014) using 50% trichloroacetic acid (CAS Number: 76-03-9) at a 1:3 w/v ratio. The homogenates were centrifuged (10,000 g, 4°C, 20 minutes) to obtain debris-free supernatants. Samples were diluted with 95% ethanol (1:3 ratio) and thoroughly mixed by repeated pipetting. Fluorescence was measured (excitation: 620 nm, emission: 680 nm) using a standard curve prepared from an EBD-albumin physiological mimic. EBD concentrations were normalized to tissue weight and expressed as μg EBD/g tissue, with final values normalized to brainstem levels.

### Statistical analysis

Statistical significances were evaluated using the Mann–Whitney U test. Correction for multiple comparisons was performed using the false discovery rate (FDR) algorithm with a q-value threshold of 0.005 for regional BBB permeability analysis. Correlation analysis was performed using Pearson’s method to examine the relationship between age and BBB permeability. The correlation coefficient (*r*), and significance level (*p*) were reported. Analyses were performed using MATLAB’s corrcoef and fitlm functions for correlation calculation and visualization, respectively. Fisher’s r to z transformation was used to compare correlation coefficients between groups.

## Results

### Regional differences in BBB permeability in the healthy human brain

We first examined regional differences in BBB permeability in healthy young individuals (Group I) by assessing median normalized BBB permeability in each lobe (see Methods). Our analysis revealed that the brainstem exhibited significantly higher median normalized permeability than seven of the nine other lobes (Fig. 1A): white matter (*p* < 0.05), cerebellum (*p* < 0.05), limbic regions (*p* < 0.05), temporal (*p* < 0.001), parietal (*p* < 0.001), occipital (*p* < 0.05), and frontal cortices (*p* < 0.001). The frontal cortex exhibited the lowest permeability, with lower median permeability than the diencephalon (*p* < 0.001) and basal ganglia (*p* < 0.0001; Fig. 1A). We next assessed BBB permeability separately in females and males. No significant sex differences in normalized BBB permeability were found (Additional Fig. 3A).

Next, we calculated fractional plasma volume (v - see Methods), reflecting vascular density, in each lobe. The frontal cortex had a higher median v compared to the brainstem, white matter, basal ganglia and diencephalon (*p* < 0.0001), suggesting greater vascular density (Fig. 1A). In both males and females, the frontal and occipital cortices displayed the highest median normalized v values, whereas the lowest values were found in the white matter and brainstem. No regional difference in v were detected between males and females (Additional Fig. 3B).

Since BBB permeability can be influenced by vascular density, we further normalized permeability measurements to plasma volume. Consistent with the results obtained for the non-normalized values, the frontal lobe maintained the lowest normalized permeability compared with the brainstem, white matter, diencephalon, basal ganglia, cerebellum (*p* < 0.0001) and the limbic lobe (*p* < 0.05, Fig. 1A). The above differences between cortical and sub-cortical regions were observed in both males and females. No differences were found between males and females in any of the lobes (for more details see Additional Fig. 3C).

### BBB permeability is not homogenous throughout the healthy rat brain

To confirm that regional differences in permeability were not due to imaging artefact (e.g. B1 inhomogeneity of the magnetic field), we tested for regional differences in permeability in rats using three independent approaches: i) DCE-MRI (n = 17, Fig. 1B), ii) fluorescent microscopy of rat brain slices following the peripheral injection of Evans blue (n = 14, Fig. 1C) and iii) spectrophotometric reading of homogenized rat brain tissue after peripheral Evans blue injection (n = 10, Fig. 1D). We found higher brainstem permeability compared to the limbic region and the isocortex using DCE-MRI (*p* < 0.001; Fig. 1B), higher brainstem permeability than the isocortex using fluorescent microscopy for brain slices (*p* < 0.001; Fig. 1C) and for homogenized brain tissues (*p* < 0.001; Fig. 1D).

BBB permeability rises with age in both sexes, more strongly in males.

**Figure 2:**
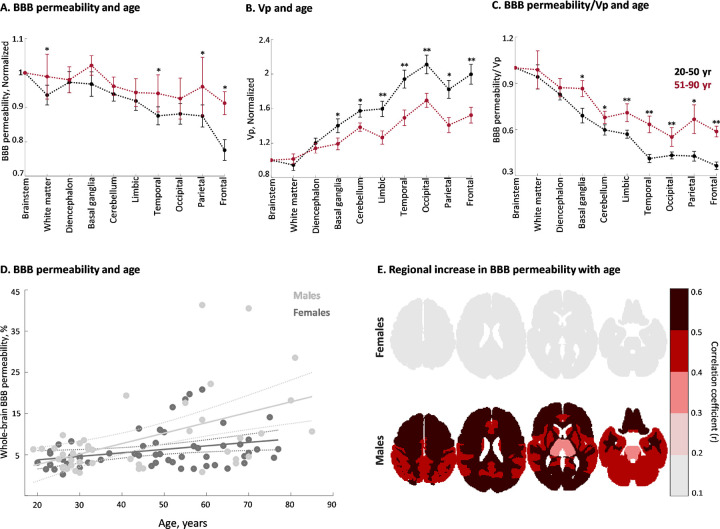
Age-related differences in BBB permeability and v across brain regions. A. Normalized BBB permeability across anatomically defined group of brain regions referred to as “lobes”, stratified by age groups (20–50 years in black; 51–90 years in red). B. Normalized plasma volume fraction (v) across the same lobes. and age groups. C. Ratio of BBB permeability to v across regions, highlighting age-related differences independent of plasma volume. Data are shown as median ± standard error. D. Association between whole-brain BBB permeability volume (%) and age, shown separately for males (light gray; Pearson correlation, *p* < 0.001, *r* = 0.51, n = 45) and females (dark gray; Pearson correlation, *p* = 0.018, *r* =0.31, n = 57). Linear regression lines with 95% confidence intervals are displayed. Each dot is an individual. Reported Pearson correlation p-values and coefficients (*r*) indicate the strength of the age association for each sex. E. Brain maps showing the Pearson correlation coefficient (*r*) between lobe-wise brain BBB permeability volume (%) and age in females (upper panel) and males (lower panel). Only lobes with Pearson correlation p-values remaining significant after correction for multiple comparisons using FDR (q = 0.05) are color-coded. Darker red reflects greater *r*. Asterisks denote Mann-Whitney U test significant differences between age groups (*p < 0.05, **p < 0.01) after correction for multiple comparisons using FDR with q = 0.005. BBB: Blood-brain barrier; FDR: False discovery rate.

We next assessed the effect of age on BBB permeability. The 102 healthy adults (19–85 years; Groups I and II, Table 1) were initially divided into three age categories (19–30, 31–50, and 51–90 years). Because BBB permeability and v did not differ between the 19–30 and 31–50 groups, these cohorts were combined for subsequent analyses. Lobe comparisons showed that individuals aged 51–90 years exhibited higher normalized BBB permeability than those aged 19–50 years in the white matter, temporal, and parietal lobes (*p* = 0.049), as well as in the frontal lobe (*p* = 0.032; Fig. 2A). In contrast, normalized v, reflecting vascularization, was higher in the younger cohort across multiple lobes, including the basal ganglia (*p* = 0.022), cerebellum (*p* = 0.002), limbic system (*p* < 0.001), temporal (*p* < 0.001), occipital (*p* < 0.001), parietal (*p* = 0.003), and frontal (*p* < 0.001) lobes, with no significant age differences observed in the white matter or diencephalon (*p* > 0.3; Fig. 2B). When accounting for vascularization older adults showed higher BBB permeability values relative to younger adults across all lobes: basal ganglia (*p* = 0.005), cerebellum (*p* = 0.005), limbic (*p* < 0.001), temporal (*p* < 0.001), occipital (*p* < 0.001), parietal (*p* = 0.002), and frontal (*p* < 0.0001), except for the white matter and diencephalon (*p* > 0.02; Fig. 2C).

Whole-brain percent volume with a leaky BBB (normalized to v ) increased with age in both males (*p* < 0.001, *r* = 0.51, n = 45) and females (*p* = 0.018, *r* = 0.31, n = 57; Fig. 2D). Lobe-wise analysis of the percent volume with a leaky BBB revealed markedly stronger age effects in males than in females, with increases observed in 10 of 10 lobes in males and none in females (Additional Table 1). Using age 30 as a data-driven threshold separated individuals into groups with different proportions of leaky voxels (both sexes combined *p* < 0.001; females *p* = 0.03; males *p* = 0.006, corrected). Below 30 years, no sex differences were detected (all *p* > 0.6). Above 30 years males (n = 27) exhibited higher permeability than males under 30 (n = 18) in all lobes, while females showed a similar trend in 2 lobes (Table 2).

### Age-related changes in BBB permeability are correlated with brain volume changes in males

Since a leaky BBB has been linked to neurodegeneration (23–26), we investigated whether age-related increase in BBB permeability is associated with decreased brain volume. Brain volume decreased with age in both males (*p* < 0.00001, *r* = −0.83, n = 45) and females (*p* < 0.0001, *r* = −0.65, n = 57; Additional Fig. 4A), with a steeper decline observed in males. In males, all lobes except the brainstem showed age-related volume reduction. In females, all lobes except the limbic system and cerebellum demonstrated age-related shrinkage (Additional Fig. 4B and Additional Table 2).

In males, linear regression analysis revealed inverse correlation between whole-brain BBB permeability and whole-brain volume (*p* < 0.001, *r* = −0.53, n = 45), suggesting that higher BBB permeability is associated with lower brain volume. However, no such correlation was found in females (*p* = 0.64, *r* = −0.06, n = 57; Additional Fig. 5A). Our lobe-based analysis identified diencephalon, occipital and frontal lobes in males where increased BBB permeability was inversely correlated with regional brain volume. In females, no lobes demonstrated such correlation (Additional Fig. 5B and Additional Table 3). These results suggest a potential association between BBB permeability and brain atrophy in aging, particularly in males, where higher BBB permeability is associated with greater regional and whole-brain volume loss.

## Discussion

Our study demonstrates that BBB permeability varies across different brain regions in both humans and rats. Cortical regions showed lower BBB permeability normalized to v for Gd-based contrast agents in humans and BBB permeability for Evans blue–albumin complex in rats compared to the brainstem and subcortical regions. We also show that BBB permeability increases with age in both females and males and the difference between older and younger individuals is more pronounced in males. Our findings suggest that age-related increase in BBB permeability is associated with a lower brain volume.

Human MRI studies have revealed BBB dysfunction in numerous pathologies including epilepsy (16, 27, 28), lupus (15), stroke (10), brain tumor (11), traumatic brain injury (5, 11, 29), Alzheimer’s disease(28, 30, 31) mild cognitive impairment(32, 33) schizophrenia (34), bipolar disorder (7), Parkinson’s disease(35) and chronic traumatic encephalopathy (31). However, most studies lack large control groups and did not account for inter-regional variations in BBB integrity within the healthy brain. To better understand BBB pathology, it is essential to obtain data on the regional, sex and age-related differences in BBB permeability in the healthy brain.

For the first time, our analysis demonstrates that BBB permeability varies across brain regions, with higher permeability observed in phylogenetically older structures compared to cortical regions, suggesting an evolutionary specialization of barrier properties occurring alongside changes in neuronal circuitry.

Notably, the most permeable regions correspond to some of the earliest structures in brain evolution. The brainstem, among the most primordial and conserved brain structures, marking the beginning of brain evolution (36), showed the highest permeability. The basal ganglia, preserved since the lamprey ~ 560 million years ago (37) and the diencephalon, likely first appearing in early chordate ~ 525 million years ago (38) also exhibited relatively high permeability. The cerebellum, emerging just before the evolution of jawed fishes ~ 450 million years ago (38, 39) displayed intermediate permeability. Limbic system, well developed in the common ancestors of reptiles and mammals (40) showed lower permeability compared to more ancient structures (41). The primary visual cortex, present in early amniotes, ~ 320 million years ago (42–45) and the neocortex, the newest addition to the mammalian brain which appeared 160–300 million years ago (46, 47), exhibited even lower permeability. Finally, the dorsolateral prefrontal cortex, a region unique to primate evolution ~ 2–3 million years ago (48), showed the lowest permeability, representing the tightest barrier.

Support for BBB development during evolution stems from a study comparing BBB structure across species, including Drosophila (fruit fly, invertebrate), sharks (primitive fish species), zebrafish (advanced teleost fish) and mammals (49). In drosophila, the BBB consists of subperineurial glia rather than endothelial cells, which is sufficient for ion regulation and preventing neural exposure to circulating hemolymph. Sharks have a glial-based BBB, where perivascular glial cells form the barrier. In zebrafish, as in mammals, capillary endothelial cells are connected by specialized tight junctions and closely interact with pericytes. While zebrafish lack astrocytes, they possess radial glial cells whose role in the BBB remains poorly characterized. In mammals, a highly restrictive BBB had evolved that is formed by tight junctions between endothelial cells and reinforced by astrocytic end-feet, pericytes, and microglia. The necessity to efficiently separate metabolic and ionic balances between the nervous system and the circulatory system accelerated the evolution of the BBB (50). Notably, large mammals exhibit a striking increase in the glia-to-neuron ratio, with humans surpassing macaques and chimpanzees (51).

Our results are consistent with previous studies showing that, in rats, the hypothalamus has the steepest leptin saturation slope and the highest brain/perfusion ratios compared to the frontal cortex (52). Similarly, in mice, the BBB in periventricular areas is less restrictive than in cortex (53). In four young, healthy individuals, Ivanidze et al. (2019) reported that the hippocampus has a higher area under the signal intensity-time curve K^trans^ compared to the orbitofrontal and prefrontal regions (54). A notable difference between the studies lies in the method to calculate BBB permeability. While Ivanidze and colleagues employed the Tofts model (54), our study measured a slow accumulation of the Gd-based contrast agent, likely indicating transcellular BBB permeability (11) (see Methods).

One key limitation of our MRI analysis in humans is the potential presence of irregularities in the distribution of the magnetic field (B1 field) across the imaging volume (55). This artifact may affect the angle at which magnetic spins are rotated away from equilibrium in the magnet, with a higher than desired angle in the center of the brain and a lower angle in the periphery (55). To confirm that the observed changes in permeability were not due to magnetic inhomogeneity we analyzed permeability differences across regions in rat brains using DCE-MRI (the smaller brain is associated with a smaller change in magnetic fields (56)) as well two methodologies to measure Evans blue-albumin complex following the systemic injection of Evans blue.

Heterogeneity in the number of neurovascular cells in various brain regions may underlie variations in BBB permeability. For example, the glia-to-neuron ration in the human cerebral cortex is 1.4, compared with 0.23 in the cerebellum (51). Pericytes also contribute to the expression of genes critical to BBB integrity, including TGFβ (57), Angiopoietin-1 (58) and PDGFβ (59). That pericyte density is lower in the spinal cord than in cortical regions (60) supports the notion that BBB is tighter in cortical regions. Furthermore, in a PDGF *β* ^ret/ret^ mouse model, BBB permeability to Evans blue or IgG was increased in the cortex, striatum, and hippocampus, while the midbrain was less affected. Interestingly, this regional heterogeneity was not driven by local pericyte coverage or tight junction integrity (61). Regional differences in the morphology of neurovascular cells may also underlie differences in barrier properties. Protoplasmic astrocytes, with their terminal processes (endfeet), which play a crucial role in BBB integrity, are highly abundant in gray matter, whereas fibrous astrocytes are mostly found in white matter (62, 63). There is also accumulating evidence that molecular and cellular variation across brain regions underlies heterogeneity in BBB permeability. Pfau and colleagues demonstrated that distinct ligand–receptor interactions between endothelial and perivascular cells, together with region-specific enrichment of tight junction and transporter gene modules, provide a mechanistic basis for regional differences in BBB tightness. In other words, the endothelial–perivascular interface appears to be differentially tuned across regions, modulating local barrier properties (64). Complementary findings by Villaseñor and colleagues, integrating in silico transcriptome analysis with in vitro and ex vivo validation, revealed that cortical microvessels are enriched in transcripts related to tight junctions, transporters, and metabolic enzymes (61). By contrast, white matter endothelium shows comparatively lower expression of barrier-strengthening genes and distinct transporter profiles (65) further supporting the concept of regional specialization in BBB molecular architecture and permeability.

Our findings further revealed regional differences in plasma volume with cortical regions showing higher v compared with sub-cortical regions (specifically white matter and brainstem). This finding aligns with previous studies showing higher vascularization of gray matter compared to white matter (66–68).

By normalizing BBB permeability to v, we attempted to distinguish increased contrast accumulation due to altered barrier properties from changes driven by vascular density. Our results showed that the regional permeability gradient is not simply explained by vascular density. The persistence of regional and age-related differences after this correction suggests that the observed effects are not solely attributable to differences in vascular volume. In fact, in younger healthy individuals cortical regions showed higher v but lower permeability, suggesting that vascular abundance and barrier restrictiveness are dissociable features of the neurovascular unit.

Our study is the first to explore age- and sex-dependent alterations in BBB permeability and vascularization using DCE-MRI. We showed that whole-brain BBB permeability increases with age in both sexes, but males are more affected. In males specifically, the frontal lobe, white matter and basal ganglia exhibited the highest age-dependent permeability increase. Our findings align with a recent arterial label signaling study of 186 cognitively normal individuals (aged 8 to 92 years) showing a decline in BBB water exchange rate (kw) with age, especially in the lateral prefrontal cortex, parietal, and lateral and medial temporal areas, with a more pronounced decrease in males compared to females (69). Rodent studies also support an age-related increase in transcellular permeability with age (12, 70).

Previous studies support the notion that age-related decrease in BBB integrity is region dependent. Using DCE-MRI in 35 cognitively normal older adults (mean age 64.5 ± 5.6 years; female: male ratio 26:9) Ha and colleagues (2021) showed that age-related BBB permeability changes are not uniform across the brain, with the hippocampus as the most vulnerable region (32), followed by the white matter, thalamus, and caudate. By contrast, frontal and parietal cortices exhibited only subtle or later changes, while the putamen showed the least evidence of permeability alterations (71). Cummins and colleagues found no evidence for age-related increase in paracellular permeability in mice (72). In contrast, studies in aged rodent models have demonstrated a shift toward increased non-specific transcellular transport across the BBB ((73); for review see (74)). Interestingly, Yang and colleagues (73) found that in young healthy mice brains, receptor mediated transcytosis is the primary mechanism for plasma protein transport across the BBB, whereas with aging, there is a shift to less selective caveolar transcytosis, along with loss of pericyte coverage. This is in line with our methodological approach for measuring BBB permeability using slow Gd accumulation, which appears to reflect transcellular transport (7).

Several mechanisms may underlie the observed increase in BBB permeability with aging. Structural remodeling of the vascular basal lamina and extracellular matrix (ECM), including stiffening, altered proteoglycan composition, and increased crosslinking, can impose mechanical strain on endothelial and perivascular cells, compromising tight junction integrity. Age-related changes in hemodynamics, such as increased pulsatility, shear stress heterogeneity, and reduced vessel compliance, further subject capillaries and microvessels to abnormal mechanical loads. Given the BBB’s sensitivity to flow dynamics, these perturbations may disrupt barrier homeostasis. In addition, weakened interactions between endothelial cells, pericytes, and the ECM in aged vessels diminish the ability to resist mechanical stress and maintain barrier tightness (75). Beyond vascular changes, age-related systemic alterations may also contribute to BBB dysfunction. Kidney function progressively declines with age, as reflected in reductions in glomerular filtration rate (76, 77). This decline impairs clearance of metabolic byproducts and toxins, which has been linked to the chronic, low-grade inflammatory state characteristic of aging, termed “inflammaging” (76). Such systemic inflammation is increasingly recognized as a key factor exacerbating BBB vulnerability. Supporting this link, Matsuki et al. demonstrated that chronic kidney disease is associated with BBB dysfunction through mechanisms involving urea-induced activation of matrix metalloproteinases and tight junction disruption (78). While these findings derive primarily from pathological conditions, they provide a mechanistic framework suggesting that age-related reductions in renal clearance may similarly contribute to BBB dysfunction by promoting systemic inflammation.

Our analysis revealed that older individuals (51–90 years) exhibited lower plasma volume compared to younger participants (19–50 years), with the most pronounced reductions in cortical regions (frontal, parietal, occipital, and temporal lobes), as well as in limbic regions and the cerebellum. These findings are partially aligned with previous studies showing a linear decline (~ 6% per decade) in the cerebral metabolic rate of oxygen (CMRO_2_), independent of atrophy, sex, or head size (79). In contrast, subcortical regions including white matter, deep gray nuclei, the thalamus, and cerebellum, showed no age-related decline in CMRO_2_. Animal experiments support these observations. In rats, in a longitudinal near-infrared spectroscopy study, cerebral blood volume decreases markedly with age and cortical blood volume declined by nearly 50% between 4 months (young adult) and 16 months (middle-aged), dropping from ~ 4.5 to 2.6 mL/100 g (80).

Our study is the first to characterize age-related BBB permeability changes while accounting for regional vascularization. These findings suggest that aging may involve both vascular rarefaction, reflected by reduced v, and increased BBB permeability. Together, this supports the concept of a dual vascular aging phenotype characterized by reduced vascular volume and increased barrier permeability relative to the remaining vascular compartment.

Our result showed a more pronounced age-dependent increase in BBB permeability of males compared to females. Experimental and review evidence suggests that estrogen contributes to maintenance of BBB integrity through regulation of endothelial function, stabilization of tight junction proteins, and modulation of inflammatory signaling pathways ((81); see review (82)). Experimental studies demonstrated that estrogen strengthens inter-endothelial junctions and reduces inflammation-induced BBB disruption (83). In contrast, age-related reductions in testosterone levels in males have been associated with vascular dysfunction and endothelial altrations during aging (84). In addition, sex-specific vascular aging mechanisms, including arterial stiffening (85), oxidative stress (86), and inflammatory vascular remodeling (87), may contribute to differential susceptibility to cerebrovascular and BBB dysfunction with aging (see review (88)). Recent imaging studies further support sex-dependent BBB aging trajectories, reporting greater BBB permeability measures in males compared to females in selected brain regions, including higher K^trans^ values in the cingulate and occipital cortices (89). Similarly, females aged 61–80 were found to have a lower CSF/serum albumin ratio compared to males, suggesting lower BBB dysfunction in aging females (90). Together, these findings suggest that sex-specific vascular and inflammatory mechanisms may contribute to the more robust age-related BBB permeability increases observed in males in the present study.

Our data suggest an association between age-related increase in BBB permeability and loss of brain volume, particularly in males. A similar association was found between the whole brain, thalamus and thalamic subregions K^trans^ values and corresponding volume in 29 schizophrenia patients (34). In contrast, no correlation was found between prior lower hippocampus volume and hippocampal BBB permeability at the time of the study (91). This suggests that hippocampal atrophy and BBB changes may occur independently or that BBB changes precede atrophy of brain cells and associated vasculature. These discrepancies highlight the importance of simultaneous measurements of vascular density and blood flow when assessing BBB permeability (see Fig. 1). Understanding the region-based, age, and sex differences in BBB permeability can inform future research, allowing for a more targeted investigation of mechanisms contributing to BBB properties across different brain regions and their alterations with aging and disease. Although increased BBB permeability was associated with lower brain volume, particularly in males, the present analysis cannot determine whether BBB dysfunction contributes to tissue loss, reflects vascular changes secondary to atrophy, or arises from shared age-related mechanisms.

Because the human data are cross-sectional, age-related differences cannot be interpreted as within-person longitudinal BBB changes. Longitudinal DCE-MRI studies will be needed to determine whether individuals with higher BBB permeability show faster progression of vascular or volumetric brain changes over time. An important implication of these findings is that BBB permeability should not be interpreted as spatially uniform across the healthy brain. The presence of regional differences, even after normalization to v, suggests that pathological BBB dysfunction should be evaluated against region-specific normative baselines. This is particularly relevant in aging and neurological disorders, where regions with intrinsically higher permeability may otherwise be misclassified as abnormal, whereas subtle changes in normally restrictive cortical regions may be overlooked. Future studies should therefore incorporate region-, age-, and sex-specific reference values when defining abnormal BBB permeability.

## Conclusion

Our study is the first to demonstrate region-based differences in BBB permeability within the healthy human brain while accounting for regional vascularization, suggesting an evolutionary specialization of barrier properties across mammalian brain regions. While an age-dependent increase in BBB permeability was observed in both males and females, it was more pronounced in males and was associated with volume loss. Understanding regional and sex-specific differences in BBB permeability may inform future research on aging and neurological disorders.

## Supplementary Files

This is a list of supplementary files associated with this preprint. Click to download.

- AdditionalFile1.docx

## Figures and Tables

**Table 1:** Demographic characteristics of the study population

	Age range	Mean age (± SD)	n female and mean age	n male and mean age
**Group I** (n = 35)	19–30	26.22 ± 3.1	n = 17 25.76 ± 3.2	n = 18 26.65 ± 3.1
**Group II** (n = 67)	31–85	56.1 ± 13.5	n = 40 56.13 ± 11.4	n = 27 56.04 ± 16.3
**Group I & II** (n = 102)	19–85	45.8 ± 18	n = 57 47.1 ± 17	n = 45 44.3 ± 18

Participants were divided into two age groups: Group I (19–30 years) and Group II (31–85 years). The table summarizes the age range, mean age ± SD, and sex distribution for each group and for the combined cohort. n: number of participants; SD: standard deviation.

**Table 2:** Age– suprathreshold BBB permeability/v summary (whole brain, hemispheres, lobes, and counts of significant regions).

Analysis level	Group(s) compared	Metric	N	r / older vs. younger	p-value	Notes
Whole brain	Age (continuous), females	Pearson *r*	57	0.33	0.013	% brain with suprathreshold BBB
Whole brain	Age (continuous), males	Pearson *r*	45	0.59	< 0.0001	
Whole brain	< 30 vs ≥ 30, all	Mann-Whitney	35 vs 67	↑ in ≥ 30	< 0.001	Same direction within each sex
Whole brain	< 30 vs ≥ 30, females	Mann-Whitney	17 vs 40	↑ in ≥ 30	0.03	
Whole brain	< 30 vs ≥ 30, males	Mann-Whitney	18 vs 27	↑ in ≥ 30	0.006	
Lobes	Correlation (top 3), females	Pearson *r*	57	-	all > 0.09	
Lobes	Correlation (top 3), males	Pearson *r*	45	Frontal 0.56; WM 0.52; Basal ganglia 0.49	all < 0.002	
Lobes	≥ 30 vs < 30, females	Mann-Whitney	17 vs 40	Limbic Parietal	both 0.002	
Lobes	≥ 30 vs < 30, males	Mann-Whitney	18 vs 27	Cerebellum	0.0006	
				Frontal	0.003	
				Brainstem	0.003	
				Basal ganglia	0.005	
				Occipital	0.008	
				White matter	0.01	
				Limbic	0.01	
				Diencephalon	0.02	
				Parietal	0.02	
				Temporal	0.02	
Hemispheres	Age (continuous), right, females	Pearson *r*	57	0.22	0.1	Between-sex difference trend for the right hemisphere *p* = 0.055 and left hemisphere *p* = 0.3 (Fisher's r to z transformation)
Hemispheres	Age (continuous), right, males	Pearson *r*	45	0.55	< 1×10^−4^	
Hemispheres	Age (continuous), left, females	Pearson *r*	57	0.27	0.038	No within-sex L vs R difference for females *p* = 0.8 and males *p* = 0. 55 (Fisher's r to z transformation)
Hemispheres	Age (continuous), left, males	Pearson *r*	45	0.46	0.001	

The table summarizes the association between age and suprathreshold BBB permeability normalized to fractional plasma volume (v) at the whole-brain, hemispheric, and lobar levels. Analyses included Pearson correlation analysis for continuous age-related associations and Mann–Whitney U tests for comparisons between younger (< 30 years) and older (≥ 30 years) individuals. Reported values include the Pearson correlation coefficient (*r*), significance level (*p*), and the number of participants (n) included in each analysis. Lobe-wise analyses highlight the brain regions exhibiting the strongest age-related associations in females and males. Hemispheric analyses additionally include comparisons between left and right hemispheres and between sexes using Fisher’s r-to-z transformation. BBB: blood-brain barrier.

## Data Availability

Raw data supporting the main and additional figures/tabels are available from the corresponding author on reasonable request. The atlas used for brain region segmentation is available at https://github.com/neurodebian/spm12/tree/master/tpm. No new software or custom algorithms were generated for data analysis for this manuscript. Analysis of blood-brain barrier permeability was performed using the BBBdetect software (Emagix Inc, NS, Canada)(7,10,15,92,93) and analysis of brain volume was performed using the Volbrain pipeline (21).

## Abbreviations

BBB: Blood-Brain Barrier
BBBdetect software: Blood-brain barrier detection software
CMRO_2_: Cerebral Metabolic Rate of Oxygen
CSF: Cerebrospinal Fluid
DCE-MRI: Dynamic Contrast Enhancing Magnetic Resonance Imaging
EBD: Evans Blue Dye
ECM: Extracellular Matrix
FDR: False Discovery Rate
FFE: Fast Field Echo
Gd: Gadolinium
Gd-DOTA: Gadoterate meglumine
GUI: Graphical User Interface
IgG: Immunoglobulin G
Ktrans: Volume transfer constant
MAD: Median Absolute Deviation
MNI: Montreal Neurological Institute
PFA: Paraformaldehyde
PDGFβ: Platelet-Derived Growth Factorβ
ROI: Region of Interest
SD: Standard Deviation
SPM: Statistical Parametric Mapping
TE: Echo Time
TGFβ: Transforming Growth Factorβ
TR: Repetition Time
v: Fractional plasma volume
